# Do other models of near-vision services compete with vision centres?

**Published:** 2026-03-12

**Authors:** Thulasiraj Ravilla

**Affiliations:** 1 Director Operations: Aravind Eye Care System, India.

**Vision centres benefit when more people become aware of near-vision spectacles.**

**Figure:**
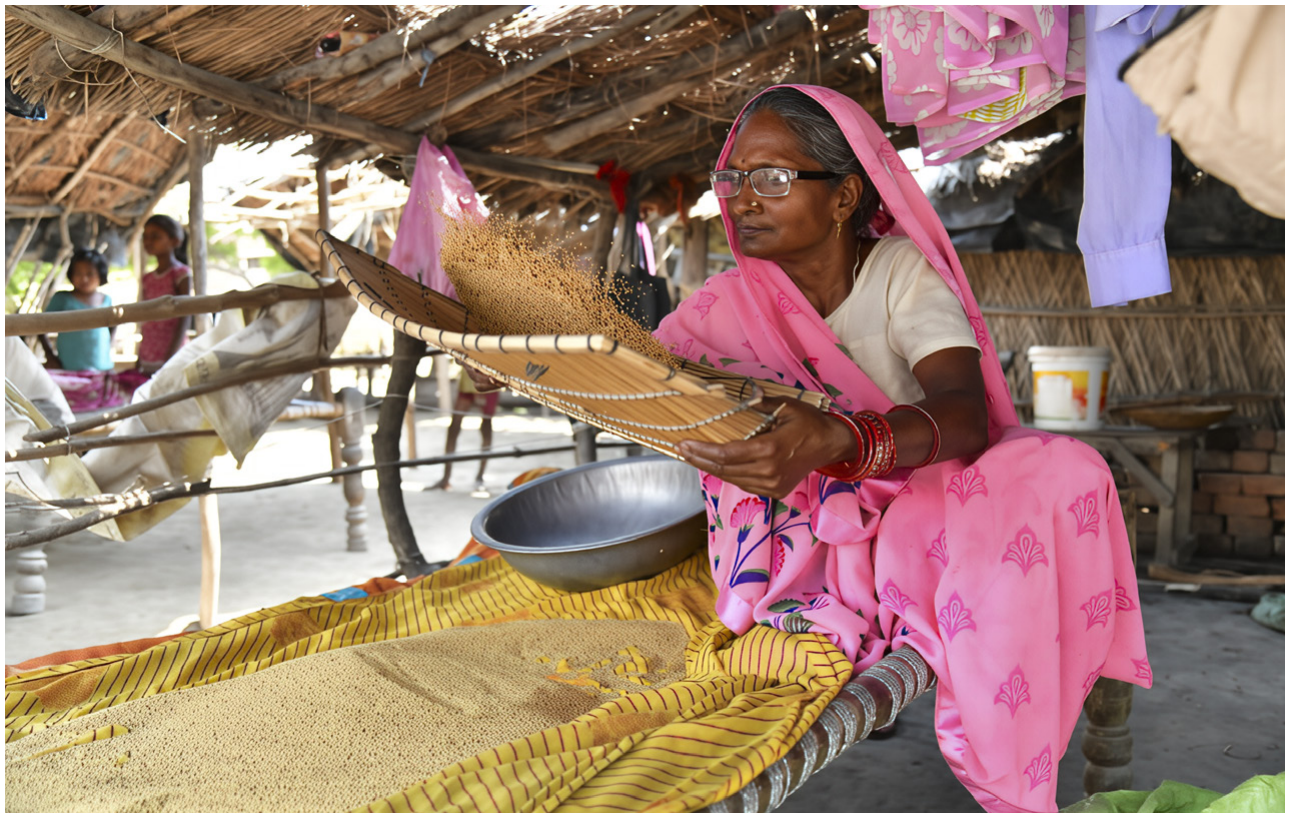
Near-vision spectacles make it easier to sift grain. INDIA

© DR SHROFFS CHARITY EYE HOSPITAL/PEEK VISION CC BY-NC-SA 4.0

There have been concerns, in India and elsewhere, that broader acceptance of non-ophthalmic approaches to the dispensing of near-vision spectacles could compete with traditional approaches, including vision centres, undermining their financial viability.

Vision centres were designed to provide primary eye care, typically serving communities living within a 10-kilometre radius. In India, this would cover a population of up to 100,000 people, depending on the population density.

It is estimated that 25-30% of India's general population have presbyopia (age-related near vision impairment) and/or distance refractive errors. Just 15% of those with presbyopia, and 35% of those with distance refractive errors, have received the spectacles they need.^1^ It is worth noting that this is a conservative estimate, based on findings from one of the better-served districts in the country.

Applying these figures to the population of 100,000 people served (on average) by a vision centre would translate to 30,000 people needing either near or distance vision spectacles. Assuming, conservatively, that the spectacles would require replacement once every four years, each vision centre would need to supply 7,500 pairs of spectacles per year.

While there is no published evidence as yet, most vision centres do not serve anywhere near this volume: current estimates of spectacle provision in vision centres average at around 1,000 pairs annually. Even at this low level of service, the margin of profit from the sale of spectacles is a significant contributor to their financial sustainability.

In India and elsewhere, there are multiple ways that people are currently able to obtain near-vision spectacles.

**Facility-based distribution.** Near-vision spectacles are dispensed in eye care facilities, such eye hospitals, optical outlets, and vision centres. Increasingly, spectacles are also being dispensed in outreach screening eye camps.

**Community-based distribution.** In the community, traditional itinerant bangle sellers, who also sold near-vision spectacles, have largely been replaced by micro-entrepreneurs with essential training who go from village to village selling ready-made spectacles for near and distance vision. The latter are supported by non-profit organisations like VisionSpring^2^ or GoodVision^3^ (formerly OneDollarGlasses) and corporate entities such as Essilor-Luxottica Foundation.^4^

**Over-the-counter distribution.** Near-vision spectacles are also sold over-the-counter in general stores and supermarkets. However, this is limited to larger urban cities.

Whether the two ‘non-ophthalmic’ distribution channels above pose a threat to the viability of vision centres, is worth contemplating.

The number of general stores or supermarkets dispensing ready-made near-vision spectacles in India is limited, and largely confined to big cities, again posing very little threat to the viability of vision centres, which are typically in small rural towns. The community-based approaches described above could overlap with the communities served by vision centres. However, these are very sporadic and - if repeated - they often take place once a year or less.

While there is clearly significant potential for vision centres to grow their refractive error services, the magnitude of the unmet need makes it unlikely that other ways of distributing near-vision spectacles - including ‘non-ophthalmic’ approaches - would compete with vision centres and existing eye care services or undermine their financial viability.

In fact, in the Indian context - where the market is very underdeveloped - the immediate strategy should be one of growing the market or increasing the number of people in need who are actually using spectacles - which to a large extent, is being done by community-based distribution and over-the-counter sales. So, these “competing” approaches are, in fact, resulting in more people becoming aware of, and using, near-vision spectacles. Because presbyopia is a life-long condition, requiring periodic replacement of spectacles, this - in reality - would further enhance the financial viability of vision centres.
